# Results of revision intramedullary nailing with and without auxillary plate in aseptic trochanteric and subtrochanteric nonunion

**DOI:** 10.1007/s00068-021-01664-6

**Published:** 2021-04-16

**Authors:** Christina Dietze, Andreas Brand, Jan Friederichs, Fabian Stuby, Dorien Schneidmueller, Christian von Rüden

**Affiliations:** 1 Department of Trauma Surgery, BG Unfallklinik Murnau, Professor Küntscher Str. 8, 82418 Murnau, Germany; 2 Institute for Biomechanics, BG Unfallklinik Murnau, Murnau, Germany; 3 Institute for Biomechanics, Paracelsus Medical University, Salzburg, Austria

**Keywords:** Nonunion, Trochanteric fracture, Subtrochanteric fracture, Femur, Cephalomedullary nail, Auxiliary plate, Augmentation, Lower Extremity Functional Scale (LEFS)

## Abstract

**Purpose:**

Aim of this study was to investigate whether limited open auxiliary angle stable plate fixation has an effect on functional and radiologic outcomes one year after revision intramedullary nailing in aseptic trochanteric and subtrochanteric fracture nonunion.

**Methods:**

In a retrospective analysis, surgically revised aseptic trochanteric and subtrochanteric nonunion was evaluated in a total of 190 consecutive patients ranging from 18 to 94 years between 12/2005 and 10/2018.

**Results:**

One year after revision intramedullary nailing, nonunion healing was assessed in 129 out of 136 patients (95%) in group 1 without auxiliary plate fixation and in 51 out of 54 patients (94%) in group 2 with auxiliary plating (*p* = 0.23). In group 1, range of motion (ROM) was unrestricted in 88 patients and still restricted in 48 patients. In group 2, ROM was free in 34 patients and restricted in 20 patients (*p* = 0.25). The mean Lower Extremity Functional Scale (LEFS) was 56 points in group 1 and 55 points in group 2 (*p* = 0.55).

**Conclusion:**

This study did not demonstrate significant differences in functional and radiologic outcomes following revision intramedullary nailing of aseptic trochanteric and subtrochanteric fracture nonunion. Limited open auxiliary plate fixation might be a reasonable option especially in cases of relevant varus axis deviation and comminuted or atypical fracture configurations, regardless of patients’ age.

Retrospectively registered with the German Clinical Trials Register (01/25/2021; ID: DRKS00024112).

## Introduction

The development of aseptic nonunion following trochanteric and subtrochanteric femoral fractures remains a major challenge for the treating surgeon. Nonunion development is based on various factors such as smoking, diabetes mellitus, peripheral vascular diseases, or lack of stability in the fracture area [1, 2], wrong indication for treatment, choice of the inadequate implant or stabilization technique, and also the incorrect follow-up treatment [3]. In this study, aseptic hypertrophic nonunion as a result of inadequate surgical stabilization and atrophic nonunion as a result of circulation problems in the fracture site and surrounding bone were investigated. Basically, nonunion development requires a careful analysis of the former fracture pattern as well as a cautious indication and an equally careful planning of the revision surgery. The main treatment goals are the restoration of stability and adjustment of the correct axial and length ratios. The current standard for the treatment of aseptic trochanteric and subtrochanteric fracture nonunion is revision intramedullary nailing aiming to change to a longer and at best large-volume nail [3–8]. The use of an intramedullary exchange nail with additional augmentation plate fixation is a well-known approach for diaphyseal femoral nonunion [8–10]. However, reliable data for trochanteric and subtrochanteric fracture nonunion despite a few case series with heterogenous patient collectives is not available [11–13].

Several studies including own biomechanical investigations in trochanteric and subtrochanteric fractures treated by cephalomedullary nailing confirmed that interfragmentary rotational and shear forces are significantly lower with the use of an auxiliary plate and that the stability of the entire construct is significantly higher than in the control group [14, 15]. The current study intended to prove this procedure also for the concept of revision intramedullary nailing in aseptic trochanteric and subtrochanteric fracture nonunion in a large number of patients. The aim was to investigate whether auxiliary limited open angle stable plate fixation has an effect on functional and radiologic results one year after revision intramedullary nail replacement.

## Methods

**Fig. 1:**
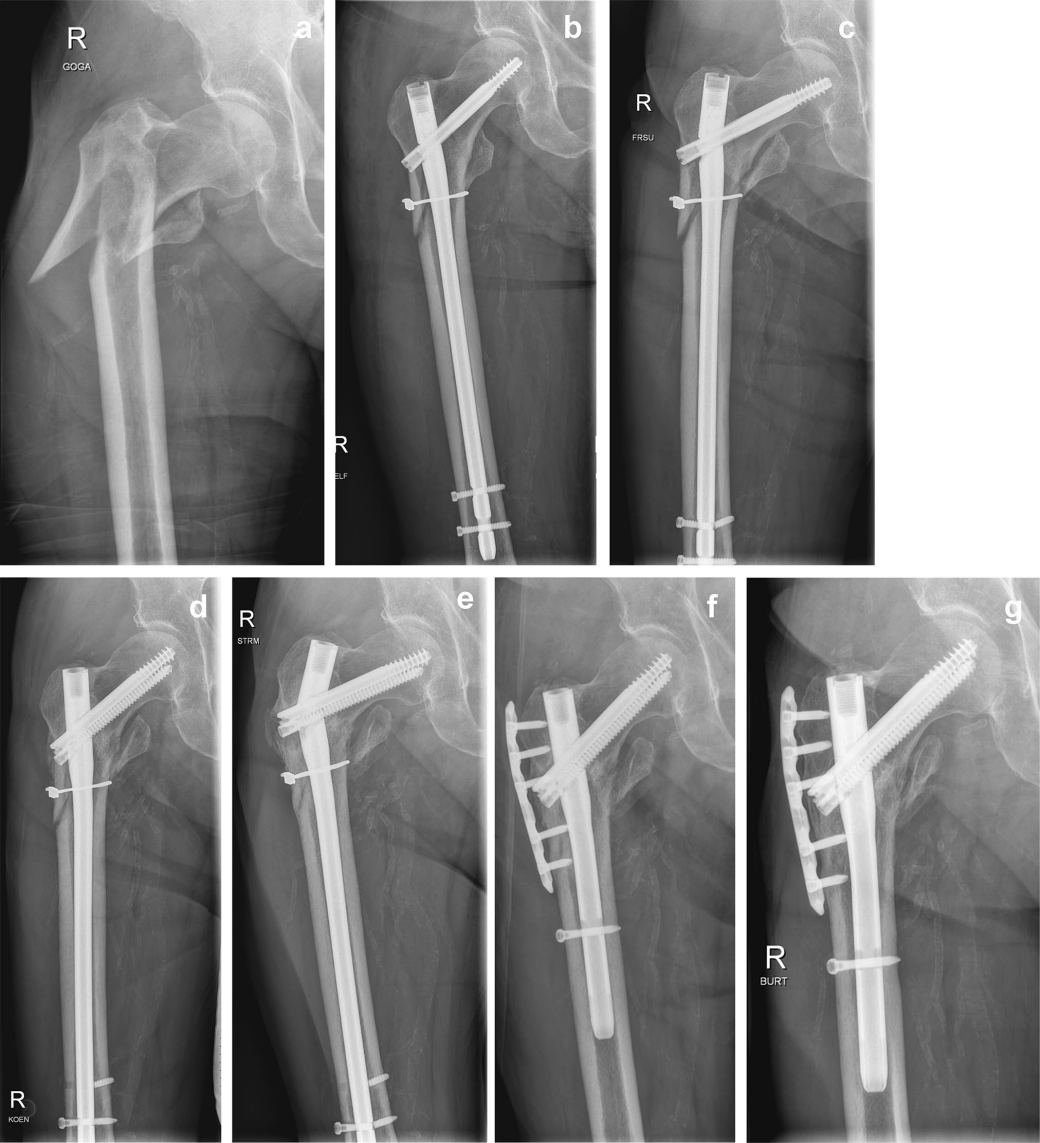
**a** 79-year-old male after fall on right hip with reversed trochanteric fracture. **b** Primary surgery including open reduction and internal fixation using the extended version of a cephalomedullary nail and auxiliary cerclage. **c** Failure of osseous healing 3 months postoperatively and breakage of the cephalomedullary nail and one of the distal locking screws. **d** Primary surgical revision using the extended version of a triangular cephalomedullary exchange nail and a renewed auxiliary cerclage following removal of the broken cephalomedullary nail and cerclage. **e** Nine months after primary surgical revision breakage of the cephalomedullary exchange nail including distal locking screw occurred. **f** Repeated revision including renewed cephalomedullary exchange nailing and limited open auxiliary angle stable plate fixation of the lateral trochanteric cortex. **g** Nonunion healing four months after repeated revision surgery

In a retrospective study, 190 consecutive patients over 18 years old with aseptic trochanteric and subtrochanteric fracture nonunion between December 2005 and October 2018 in a European level 1 trauma center were included. Revision surgery was performed using intramedullary nail replacement without auxiliary plate fixation in group 1 and with the use of limited open auxiliary plate fixation in group 2 (Fig. 1a–g).

### Diagnostic work-up prior to revision surgery

Only patients without clinical signs of local infection and without any evidence of infection in the anamnesis were recorded. Clinical signs of aseptic nonunion included an inability to perform a full weight load without pain or persistent instability in the fracture area. Radiologic signs of nonunion were defined as a lack of bony bridging in at least three of four cortices assessed on conventional radiographs in antero-posterior and lateral views. If conventional radiographs were inconclusive to diagnose nonunion, a computed-tomography (CT) scan was performed to determine the presence of nonunion. Preoperative biplanar radiographs and CT scans, and intraoperative, postoperative radiographs as well as the complete medical report were documented. The preoperative work-up for infection also consisted of blood samples including C-reactive protein and white blood cell values. Coding of the initial fracture was done independently by two senior orthopaedic surgeons. The AO/OTA classification (type 31 A1–3) was used for trochanteric fractures and the Seinsheimer classification (grade 1–5) for subtrochanteric fractures [16]. The currently accepted definition describes nonunion as a fracture that will not heal without further medical intervention regardless of the duration of treatment [1]. Nonunion was differentiated into atrophic and hypertrophic according to the radiographic pattern. Besides, identification of the previous implant and the potential need for any special equipment required for removal was conducted.

### Surgical procedure

All surgical procedures were performed in a standardized manner under image intensifier control with patients in a lateral position on a radiolucent operating table according to the instructions provided by Friederichs et al. [17]. The surgical measures as well as the procedure and selection of implants were comparable in both treatment groups. The inserted fixation material was removed through the present approach. In the case of a varus or flexion deviation, the axis was corrected via the new implant. In each case, the medullary canal was reamed. If no new entry point was required, the same entry canal was overreamed slightly more posteromedially. The overreaming of the nonunion area was accomplished stepwise. A revision intramedullary nail as long and thick as possible was applied. The inserted revision nail was used in both treatment groups according to manufacturer's instructions (Gamma3 Nailing System, Stryker Corp., Kalamazoo, MI, USA; TRIGEN INTERTAN, Smith & Nephew Inc., Memphis, TN, USA). The revision implant in each case was selected by the treating surgeon. The aim was (1) to avoid the position of the previous intramedullary nail and (2) to improve stability by positioning the revision nail in stable bone depending on type and position of the previous nail. The nonunion area was debrided via limited exposure, followed by subsequent internal fixation using an angle stable 3.5 mm small fragment plate (LCP, Synthes GmbH, Oberdorf, Switzerland) [18]. The optional use of additional autologous cancellous bone harvested from the ipsilateral iliac crest was dependent on the discretion of the treating surgeon. It was based on the size and extent of the bone loss and the vitality and quality of the tissue in the nonunion area.

Intraoperatively, tissue samples were collected for histopathological and microbiological examination either from the reaming debris in group 1 or directly from the nonunion tissue in group 2 to exclude an inflammatory cause for nonunion development [19]. After sample collection, a single prophylactic antibiotic administration was applied based on the clinic protocol. Aftercare was conducted using the same rehabilitation protocol allowing early weight bearing in all patients [20]. Septic nonunion, nonunion based on pathologic or periprosthetic fractures, and nonunion treated by an external fixation procedure were excluded from this study. Besides, patients under 18 years of age and patients unable to provide written informed consent were excluded.

### Follow-up

Patients were followed-up functionally and radiologically at 6 weeks, 3 months and 12 months after surgical revision in the outpatient department of the hospital. Functional outcome and patient satisfaction were observed using the Lower Extremity Functional Scale (LEFS) [21]. This score consists of 20 items, each with a maximum score of 4 points. The total possible score of 80 points indicates a high functional level of the lower extremity. Range of motion (ROM) of the hip joint was assessed using the neutral zero passage method [22]. Radiologic follow-up was assessed using anterior–posterior and lateral radiographs. Bone healing was defined as the ability to bear full weight without pain, stability at the former nonunion site, formation of bridging callus at all four cortices, and absence of fracture lines [23]. The cervicodiaphyseal angle (CDA) was measured between the femoral shaft axis and the femoral neck axis, normally ranging between 110° and 140°, with an average of 128° (female 127°, male 132°). Varus axis deviation is associated with an angle less than the normal range, and valgus axis deviation with an angle greater than the normal range.

### Statistical analysis

Data were managed using Excel® for Windows® (Microsoft Corp., Redmond, WA, USA). IBM SPSS® Statistics for Windows (IBM Corp., Armonk, New York, USA) was used for statistical analysis. Test for normal distribution was performed using the Shapiro–Wilk test. Chi-squared test (gender, type of nonunion, nonunion healing, ROM, implant failure), Mann–Whitney test (Fracture type, radiographic classification, LEFS) and Student’s *t* test (age, time between initial and revision surgery) were used for statistical analysis. A result was considered to be statistically significant with *p* value < 0.05.

## Results

Patients’ basic data are presented in Table 1. Group 1 included 136 out of 190 patients (33 female, 103 male), group 2 included the remaining 54 patients (17 female, 37 male). Mean age of patients was 45.3 ± 16.3 years in group 1 versus 56.4 ± 17.7 years in group 2 and ranged from 18 to 94 years. In 135 out of 190 patients, initial fracture care was performed in an outside institution, whereas in the remaining 55 patients, initial treatment was carried out at our hospital.

**Table 1:** Comparison of patients’ general data between the treatment groups (data presented as ratios or as mean ± standard deviation)

Parameter	Group 1 (*n* = 136)	Group 2 (*n* = 54)
Age (years)	45.3 ± 16.3	56.4 ± 17.7
Gender (female/male)	33/103	17/37
Fracture type (trochanteric/ subtrochanteric)	43/93	34/20
Trochanteric fracture (AO/OTA A1/ A2/ A3)	9/20/14	9/12/13
Subtrochanteric fracture (Seinsheimer Grade 1/2a/2c/3a/3b/4)	29/12/12/17/17/6	12/1/0/3/4
Type of nonunion (hypertrophic/atrophic)	117/ 19	50/ 4
Range of time between initial and revision surgery (months)	11.5 ± 8.4	16.2 ± 15
CDA prior to revision surgery (degree)	124 ± 3.1	123 ± 2.1
CDA after revision surgery (degree)	125 ± 2.6	130 ± 4.8

According to the AO/OTA classification, group 1 demonstrated a distribution pattern of 9 A1, 20 A2, and 14 A3 fractures. According to the Seinsheimer classification, 29 Grade 1, 12 Grade 2a, 12 Grade 2c, 17 Grade 3a, 17 Grade 3b, and 6 Grade 4 fractures were coded. In 86 cases, a single cephalomedullary nail was used in the initial procedure. Seventeen patients received a cephalomedullary nail with additional cerclage. Nineteen fractures were treated initially using an antegrade femoral nail with additional cerclage. In seven patients, a retrograde nail was used. In four cases, fractures were fixed by Dynamic Hip Screw (DHS), in two cases by a curved condylar plate, and in one case by an angled blade plate. In group 1, nonunion was classified as hypertrophic in 117 cases and as atrophic in 19 cases.

In group 2, 9 patients had an A1 fracture, 12 patients an A2 fracture, and 13 patients an A3 fracture. According to the Seinsheimer classification, 12 Grade 1, 1 Grade 2a, 3 Grade 3b, and 4 Grade 4 fractures were coded. In group 2, 20 patients were treated initially using a cephalomedullary nail. In 19 patients, an auxiliary cerclage was applied to the intramedullary nail. Seven fractures were treated using an antegrade femoral nail with additional cerclage. A retrograde nail was used in three patients, and a DHS in two patients. Two patients were treated by a curved condylar plate and one remaining patient by an angled blade plate. In group 2, nonunion was classified as hypertrophic in 50 patients and as atrophic in four patients.

Prior to surgical revision, in group 1, 20 patients reported permanent pain, 101 patients reported pain on exertion, and 15 patients were not able to move without walking aids. In group 2, 19 patients described permanent pain, 32 patients reported pain on exertion, and 3 patients were able to move only with the use of crutches before revision surgery.

Revision intramedullary nailing without auxiliary plate fixation in group 1 was performed after 11.5 ± 8.4 months following initial fracture stabilization using an extended cephalomedullary nail in 114 patients (91 × Gamma3 280–440 mm; 23 × TRIGEN INTERTAN 260–460 mm). In 7 patients, an antegrade femoral nail (T2 GTN, Stryker Corp., Kalamazoo, MI, USA) and in 15 patients an extended cephalomedullary nail with supplemental cerclage was used. Supplemental osteoinductive autologous cancellous bone from the iliac crest was applied in 30 patients. Twenty-five hypertrophic and 5 atrophic nonunion cases were found in these 30 patients.

In group 2, intramedullary nail replacement combined with auxiliary plate fixation was performed 16.2 ± 15 months following initial surgical fracture treatment (*p* = 0.04). Auxiliary plate fixation without replacement of the initial intramedullary nail was carried out in 10 patients [9]. In 33 patients, the previously inserted nail was replaced using an extended Gamma3 cephalomedullary nail. T2 GTN antegrade femoral nailing was used in the remaining 11 patients. Autologous cancellous bone graft was additionally applied in 36 patients, in 34 cases of hypertrophic and in 2 cases of atrophic nonunion.

The mean CDA was 124° ± 3.1° preoperatively versus 125° ± 2.6° postoperatively with a mean valgus correction of 1° in group 1 (*p* = 0.072) and 123° ± 2.1° preoperatively versus 130° ± 4.8° postoperatively with a mean valgus correction of 7° in group 2 (*p* = 0.001) (Table 1).

One year after surgical revision, radiologic follow-up examination of group 1 demonstrated nonunion healing in 129 out of 136 patients (95%). In seven patients (3 × AO/OTA 31A1, 1 × 31A2, 2 × 31A3, 1 × Seinsheimer 2a), there was no healing tendency. Fifteen patients underwent one or more additional revision surgeries in the postoperative course due to implant failure or persistent pain (Table 2). In group 2, nonunion healing was assessed radiologically in 51 out of 54 patients (94%; *p* = 0.23; Table 2). In three remaining patients (1 × AO/OTA 31A3, 1 × Seinsheimer 2a, 1 × Seinsheimer 3b), there was no healing tendency. In this group, the fixation material had to be replaced once again in nine patients due to implant failure or persistent pain (*p* = 0.29).

**Table 2:** Comparison of functional and radiologic 1-year follow-up between the treatment groups (data presented as ratios or as mean ± standard deviation)

Parameter	Group 1 (*n* = 136)	Group 2 (*n* = 54)	*P* value
Radiologic nonunion healing (yes/no)	129/7	51/3	0.23
ROM [22] (free/restricted)	88/48	34/ 20	0.25
LEFS [21] (points)	56 ± 20	55 ± 19	0.55
Revision implant failure (yes/no)	15	9	0.29

In the final follow-up, functional outcomes correlated with these results (Table 2): In group 1, unrestricted ROM of the hip joint was achieved in 88 patients and was still restricted in 48 patients. In group 2, ROM was free in 34 patients and still restricted in 20 patients (*p* = 0.25). Functional outcome according to the LEFS demonstrated 56 points in group 1 compared with 55 points in group 2 (*p* = 0.55).

## Discussion

The aim of the present study was to compare functional and radiologic results following surgical revision of aseptic trochanteric and subtrochanteric nonunion using intramedullary nail replacement with and without auxiliary plate fixation. The results of this study demonstrated comparable very good radiologic healing rates in both treatment groups. Encouragingly, good functional outcome and patient satisfaction were evaluated for both treatment groups, independent of patients’ age.

Basically, several biomechanical peculiarities for the treating surgeon have to be considered, which may affect fracture healing at the proximal femur. First, there is significant varus stress on the proximal femur under loading, and second, it is largely composed of cortical bone, which is why osseous union is achieved slowly [24]. For these and other reasons, trochanteric and subtrochanteric fractures in particular are more at risk of developing nonunion than other fractures. Previous studies reported a nonunion rate of up to 20% for unstable trochanteric and subtrochanteric fractures and described that intramedullary force carriers were able to achieve higher rates of osseous healing with lower complication rates than extramedullary implants [3]. Revision intramedullary nailing has been established as a superior surgical approach for the treatment of aseptic trochanteric and subtrochanteric fracture nonunion due to the interplay of multiple causes including malalignment, bone loss, implant breakage, and lack of blood supply [25]. However, there is still a lack of agreement, even for the method of intramedullary nail replacement, as to how this approach to the problem is best accomplished in detail.

Whereas in femoral shaft fractures healing usually depends on axial position and loading as an impulse for osseous union, the situation is different for unstable trochanteric and subtrochanteric fractures. Here, the goal of surgical therapy is to give the fracture as much stability as possible in the correct axial and rotational position to allow fracture healing. Therefore, nonunion revision is aiming slight valgization with an anatomical rotational position. Limited open auxiliary angle stable plate fixation of the lateral cortex has proven to be a complementary tool to the revision concept of revision intramedullary nailing. In contrast to the femoral diaphysis, where gradual reaming of the medullary canal is the decisive factor of the revision concept [26, 27], the distance between the nail entry point and the nonunion area on the proximal femur is too short. In this context, angle stable auxiliary plate not only has the task of securing reduction, but also of increasing overall stability of the whole static construct in the correct axial and rotational position [28].

The time between surgical revision and nonunion healing did not reveal significant differences. A larger correction value was recognizable in the group with auxiliary plate than in the group without plate. In so far, a trend could be observed that the more complex fracture nonunion cases were treated in the group with auxiliary plate. There were no significant differences between the study groups in terms of complications. This fact suggests that although auxiliary plate fixation is a supplementary intraoperative measure, it may not lead to increased complication rates. Furthermore, there was no discernible relationship between healing rates and nonunion type. This might suggest that the use of an auxiliary plate may depend more on the decision of the treating surgeon, based on his or her functional experience, than on the definitive indication. Since the use of an auxiliary plate in this study was not associated with increased complication rates, its age independent use can be recommended despite the slightly increased intraoperative effort especially in cases of comminuted or atypical fracture pattern or in cases of limited possibility of axis and rotation correction during revision nailing.

Nevertheless, the study has some limitations such as its retrospective nature. Accordingly, it was not possible to randomize age, gender and indication for the additional use of an auxiliary plate. Advantages are the exceedingly large cohort size and the fact that all patients were treated by the same team of surgeons in the same clinic according to the same treatment and aftercare protocol. Considering that even the cases of subtrochanteric nonunion are very rare and difficult to collect and that only few cases are available in literature at all, the results of this study with a follow-up of consecutive patients are significant.

## Conclusion

Functional and radiologic results following surgical revision of trochanteric and subtrochanteric aseptic nonunion using intramedullary nail replacement with and without limited open auxiliary angle stable plate fixation were good in this large patient cohort. Overall, significant differences between intramedullary nail replacement alone and the use of auxiliary plating could not be assessed. Nevertheless, this procedure might be a reasonable option especially in cases of relevant varus axis deviation and comminuted or atypical fracture configurations, regardless of patients’ age.

## Data Availability

The datasets analyzed during the current work are available from the corresponding author upon reasonable request.

## References

[1] Perren SM, Fernandez A, Regazzoni P (2015). Understanding fracture healing biomechanics based on the "strain" concept and its functional applications. Acta Chir Orthop Traumatol Cech.

[2] Augat P, Hollensteiner M, von Rüden C (2020). The role of mechanical stimulation in the enhancement of bone healing. Injury.

[3] Dziadosz D (2015). Considerations with failed intertrochanteric and subtrochanteric femur fractures: how to treat, revise, and replace. J Orthop Trauma..

[4] Barquet A, Mayora G, Fregeiro J, López L, Rienzi D, Francescoli L (2004). The treatment of subtrochanteric nonunions with the long gamma nail: twenty six patients with a minimum 2-year followup. J Orthop Trauma.

[5] Haidukewych GJ, Berry DJ (2004). Nonunion of fractures of the subtrochanteric region of the femur. Clin Orthop Relat Res.

[6] Kempf I, Grosse A, Rigaut P (1986). The treatment of noninfected pseudarthrosis of the femur and tibia with locked intramedullary nailing. Clin Orthop Relat Res.

[7] von Rüden C, Hungerer S, Augat P, Trapp O, Bühren V, Hierholzer C (2015). Breakage of cephalomedullary nailing in operative treatment of trochanteric and subtrochanteric femoral fractures. Arch Orthop Trauma Surg.

[8] Webb LX, Winquist RA, Hansen ST (1986). Intramedullary nailing and reaming for delayed union or nonunion of the femoral shaft. A report of 105 consecutive cases. Clin Orthop Relat Res..

[9] Ueng SW, Chao EK, Lee SS, Shih CH (1997). Augmentative plate fixation for the management of femoral nonunion after intramedullary nailing. J Trauma.

[10] Roetman B, Scholz N, Muhr G, Möllenhoff G (2008). Augmentive plate fixation in femoral non-unions after intramedullary nailing. Strategy after unsuccessful intramedullary nailing of the femur. Z Orthop Unfall..

[11] Lo YC, Su YP, Hsieh CP, Huang CH (2019). Augmentation plate fixation for treating subtrochanteric fracture nonunion. Indian J Orthop.

[12] Birjandinejad A, Ebrahimzadeh MH, Ahmadzadeh-Chabock H (2009). Augmentation plate fixation for the treatment of femoral and tibial nonunion after intramedullary nailing. Orthopedics.

[13] Benz D, Tarrant SM, Balogh ZJ (2020). Proximal femur fracture non-union with or without implant failure: a revision technique with functional outcomes. Injury.

[14] Eberle S, Gabel J, Hungerer S, Hoffmann S, Pätzold R, Augat P, Bühren V (2012). Auxiliary locking plate improves fracture stability and healing in intertrochanteric fractures fixated by intramedullary nail. Clin Biomech (Bristol, Avon).

[15] Wang ZH, Li KN, Lan H, Wang XD (2020). A comparative study of intramedullary nail strengthened with auxiliary locking plate or steel wire in the treatment of unstable trochanteric fracture of femur. Orthop Surg.

[16] Loizou CL, McNamara I, Ahmed K, Pryor GA, Parker MJ (2010). Classification of subtrochanteric femoral fractures. Injury.

[17] Friederichs J, von Rüden C, Hierholzer C, Bühren V (2015). Antegrade femoral intramedullary nailing in a lateral position. Unfallchirurg.

[18] Palm H, Jacobsen S, Sonne-Holm S (2007). Hip fracture study group. Integrity of the lateral femoral wall in intertrochanteric hip fractures: an important predictor of a reoperation. J Bone Joint Surg Am..

[19] Stucken C, Olszewski DC, Creevy WR, Murakami AM, Tornetta P (2013). Preoperative diagnosis of infection in patients with nonunions. J Bone Joint Surg Am.

[20] Lizano-Díez X, Keel MJB, Siebenrock KA, Tey M, Bastian JD (2020). Rehabilitation protocols in unstable trochanteric fractures treated with cephalomedullary nails in elderly: current practices and outcome. Eur J Trauma Emerg Surg.

[21] Binkley JM, Stratford PW, Lott SA, Riddle DL (1999). The Lower Extremity Functional Scale (LEFS): scale development, measurement properties, and functional application. North American Orthopaedic Rehabilitation Research Network. Phys Ther..

[22] Seyfarth H (1974). Principles of the neutral-zero-passage method. Beitr Orthop Traumatol.

[23] Fisher JS, Kazam JJ, Fufa D, Bartolotta RJ (2019). Radiologic evaluation of fracture healing. Skeletal Radiol.

[24] Hollensteiner M, Sandriesser S, Bliven E, von Rüden C, Augat P (2019). Biomechanics of osteoporotic fracture fixation. Curr Osteoporos Rep.

[25] DeRogatis MJ, Kanakamedala AC, Egol KA (2020). Management of subtrochanteric femoral fracture nonunions. JBJS Rev.

[26] Verma R, Sharma P, Gaur S (2017). Augmentation plating in management of failed femoral nailing. Injury.

[27] Perl M, Hierholzer C, Woltmann A, Bühren V (2016). Exchange intramedullary nailing technique for aseptic hypertrophic femoral shaft pseudarthrosis. Trauma Berufskrankh.

[28] Vaishya R, Agarwal AK, Gupta N, Vijay V (2016). Plate augmentation with retention of intramedullary nail is effective for resistant femoral shaft non-union. J Orthop.

